# Leaf gas exchange and δ^13^C in cowpea and triticale under water stress and well-watered conditions

**DOI:** 10.1016/j.heliyon.2021.e07060

**Published:** 2021-05-21

**Authors:** Lawrence Munjonji, Kingsley Kwabena Ayisi

**Affiliations:** University of Limpopo, Risk and Vulnerability Center, P Bag X1106, Sovenga, 0727, South Africa

**Keywords:** Leaf gas exchange, Water stress, Carbon isotope composition, C3, Triticale, Cowpea

## Abstract

Leaf gas exchanges play a critical role in determining crop productivity as they control both CO_2_ gain and water loss. CO_2_ gain and water loss influence water use efficiency (WUE) and carbon isotope composition (δ^13^C). Responses in leaf gas exchanges to water stress are species-specific. However, the extent of this variation in C3 crops is less studied. A field study was carried out to investigate the influence of water stress on leaf gas exchanges of triticale and cowpea. Crops were grown under water stress and well-watered conditions and leaf gas exchanges were determined at flowering. The results showed that triticale maintained a higher stomatal conductance (*gs*), transpiration rate(*E*) and intercellular CO_2_ concentration (*ci*) compared to cowpea but did not differ in photosynthetic rate(*A*). As a result, triticale discriminated against ^13^C more than cowpea. These results suggest a higher influence of *ci* on δ^13^C than *A*. Despite triticale maintaining higher rates of *ci*, *A* and *gs*, it had lower WUE compared to cowpea. Consequently, triticale grain yield was more sensitive to water stress than cowpea. The findings of this study showed significant variation in leaf gas exchanges and δ^13^C between two drought-tolerant C3 crops suggesting differences in their response mechanism to water stress.

## 1 Introduction

Drought is the major abiotic restriction to crop productivity and is expected to become progressively severe and more frequent due to climate change. Drought has a significant influence on food security, particularly in regions where crop production solely dependents on rainfall. Drought is a major problem in arid and semi-arid areas where rainfall is very low. As a result of climate change and variability, rainfall in many of these areas is predicted to decrease and become even more erratic. Global warming is also expected to decrease soil moisture through increased evapotranspiration and thus inhibit plant growth [1].

Cowpea (*Vigna unguiculata*) is an essential C3 legume crop, commonly produced in tropical and subtropical dry areas of the world where the production usually depends on rain as the only source of water supply [2]. Cowpea does not only enhance soil fertility when the stover is retained, but is also an important protein source, mainly to the poor rural populations. Triticale (x. *Triticosecale* Wittmack) is a C3 small grain cereal that grows vigorously with many possible food and feed uses in the future. Triticale yields high; is highly tolerant to many pests and diseases and is adaptable to poor growing conditions [3].

Water stress (resulting from drought) induces a series of morphological, physiological, biochemical, and molecular responses [2]. Some common morphological responses to water stress include reduction in leaf size, and stunting [4, 5]. Leaf gas exchanges such as *gs, E* and *A* are some of the core physiological responses to drought. When plants experience mild drought, their immediate response is a decrease in stomatal conductance which in turn reduce water loss and consequently limits photosynthesis rate. When water stress is severe, the photosynthesis rate (CO_2_ assimilation) is not only limited by stomatal conductance but also by non-stomatal limitations like mesophyll conductance and biochemical limitation [1, 2].

The reduction in CO_2_ assimilation or in the concentration of intercellular CO_2_ affects ^13^C discrimination. Higher intercellular CO_2_ concentration promotes ^13^C discrimination while the reduction in CO_2_ concentration will result in less discrimination.

As a result, more negative δ^13^C are observed at higher intercellular CO_2_ concentration. Values of δ^13^C are also influenced by the photosynthesis pathway of the plant. More negative values are naturally observed in C3 plants than in C4 as a result of the differences in carbon dioxide affinity of the fixing enzymes. Ribulose bisphosphate carboxylase/oxygenase (RUBISCO) is the enzyme used for CO_2_ fixation in C3 plants and is less efficient in fixing CO_2_ when compared to Phosphoenolpyruvate carboxylase (PEP) in C4 plants. As a result, C4 plants incline to having higher water use efficiency (WUE) compared to C3 plants. Even among genotypes of the same species, genotypes with less negative δ^13^C tend to have higher WUE. Some studies have employed the use of δ^13^C to select high performing wheat genotypes under limited water supply [6].

Understanding plant leaf gas exchange and how they respond to water availability is very important not only for cultivar selection but also for future breeding purposes in the face of climate change. It's also important to understand how plants with diverse photosynthetic pathways and leaf morphologies respond to water stress. Many studies on leaf gas exchanges are normally restricted to the comparison of varieties of the same species [7, 8, 9, 10]. A few studies compare species in the same family, for example, *Gramineae* [11, 12] or *Leguminosae* [13]. Studies assessing the influence of different moisture levels on leaf gas exchanges are even scantier.

The aim of this study was out to evaluate the difference in leaf gas exchanges and carbon isotope composition of a C3 legume (cowpea) and a C3 cereal (triticale) to two distinct moisture levels. We hypothesized that due to the inherent drought tolerance of these two crops, there is no variation in leaf gas exchange and carbon isotope composition response to soil moisture level.

## 2 Methodology

### 2.1 Crop growth conditions

Field experiments were carried out at the experimental farm of the University of Limpopo, Syferkuil, Limpopo Province, South Africa. Syferkuil has temperatures ranging from 17 to 27 °C in summer and 4–20 °C in winter. Soils at Syferkuil were classified according to WRB [14] as Chromic Luvisols. Topsoil (0–30cm) were sampled and analyzed before planting each crop and fertilization was adapted to each crop requirement (see below sections).

The experiment was established as a randomized complete block design in split-plot arrangement where moisture level constituted the main plot treatment and triticale and cowpea genotypes, the subplot treatment. Four genotypes of each crop were used. The four triticale genotypes were used in this study were Baccus, Agbeacon, Rex and US2007. Similar to triticale, four genotypes of cowpea were also used in the current study. The genotypes were TVu4607, IT99K-1122, IT00K-529-1 and TVu4607. Two moisture levels comprising the following were assessed:

• Well-watered (WW): allowing 25% soil moisture to be depleted before irrigating back to field capacity (FC);
• Water stressed (WS): For cowpea, the crops depended solely on rainfall (rainfed) as it was carried out during the rainy season. For triticale, the plots were first irrigated to FC and then was allowed to dry out, with supplementary irrigation of 40mm later in the season.

All treatments were replicated four times. The sizes of the plots were 100m^2^ irrigated by sprinklers fitted with water meters to measure irrigation water. Each plot was also fitted with rain gauges to confirm the amount of water applied.

#### 2.1.1 Triticale

Triticale was mechanically planted in winter months from June to October in 2013 in rows, 25 cm apart at an approximate plant population of 200 plants square meter. Biomass was determined at early milking stage and grain yield at harvesting. The biomass was collected from inner rows of the plots by incising plants at 10 cm above the soil surface and oven drying it at 65 °C until constant weight. Leaf gas exchanges measurement were carried out at flowering from the midrib of the flag leaf as described under section 3.2. Flag leaves were also sampled for δ^13^C analyses (section 3.3).

#### 2.1.2 Cowpea

Cowpea was planted in the 2014/2015 rainy season from December to April in rows that were 0.9 m wide and intra-row seeds spaced at 0.20 m. Cowpea was sown with no inoculation and thus relied on the *Bradyrhizobia* existing in the soil for nodulation. Aboveground biomass was sampled from an area of 0.9 m^2^ after flowering of 50% of the cowpea. The aboveground biomass was determined by incising the main stalk at 3 cm above the soil surface and was then oven dried at 65 °C. Grain yield was harvested from inner rows that were 2 m long. Leaf gas exchange measurements were carried out on the youngest fully matured and illuminated leaf (section 3.2). The leaves used when measuring gas exchanges were later sampled for δ^13^C analyses (section 3.3).

### 2.2 Leaf gas exchanges

Leaf gas exchange such as intercellular CO_2_ concentration (*ci*), photosynthetic rate (*A*), transpiration rate (*E*) and stomatal conductance (*gs*) were measured using LCi-SD Ultra-Compact Photosynthesis System (ADC Bio Scientific, UK). All the measurements were done on cloud free days between 10h00 and 14h00. Leaf gas exchanges were measured at the flowering stage of each crop.

### 2.3 Carbon isotope composition analyses (δ^13^C)

Leaves sampled for isotope analyses were oven dried at 65 °C first and then milled using a ZM200 mill (Retsch, Germany). Carbon isotope composition was analysed by an Automated Nitrogen Carbon Analyzer (ANCA-SL, SerCon, UK) connected to an Isotope Ratio Mass Spectrometer (IRMS) (20-20, SerCon, UK). The isotope signatures were reported as δ^13^C in per mil using Vienna Pee Dee Belemnite (V-PDB) as an international standard (R_standard_) and calculated using Eq. (1)

(1) $$\delta^{13}{\text{C}}_{\text{sample}}=\left(\frac{{\text{R}}_{\text{sample}}}{{\text{R}}_{\text{standard}}}-1\right)\times 1000$$

### 2.4 Data analyses

SPSS 20 was used for data was analyses. The General Linear Model was used in the analyses. Where significant differences were observed mean separation was done using Tukey. Correlations were also carried out to measure the relationships between parameters.

## 3 Results and discussion

### 3.1 Total irrigation water and weather conditions at leaf gas exchange measurement

Table 1 shows the different amounts of water received by the two crops under WW and WS conditions along with the weather conditions when gas exchange measurements were taken. The amount of water received by cowpea under WW was 65% more than what was received under WS. In the triticale experiment, well-watered conditions received 50% more water compared to water-stressed conditions. Leaf gas exchanges at leaf level are generally influenced by temperature, light and vapour pressure deficit (VPD). Table 1 shows temperature and VPD recorded on the same day leaf gas exchanges were measured. On both occasions (i.e. for cowpea and triticale) the VPD was 1.1 kPa. The maximum temperature was relatively similar but minimum temperature was low for triticale.

**Table 1:** Water received and weather conditions experienced on the day gas exchange measurements were taken for both cowpea and triticale.

	Cowpea	Triticale
**Moisture level**		
Well-watered (mm)	348	450
Water stressed (mm)	121	226
Percentage difference	65	50
**Weather conditions**		
Minimum T (°C)	11	4
Maximum T (°C)	24	23
VPD (kPa)	1.1	1.1
Minimum RH (%)	33.1	31.5
Maximum RH (%)	82.4	83.6
Radiation (MJ/m^2^)	27.4	20.8

VPD is vapour pressure deficit, RH is relative humidity, T is temperature.

### 3.2 Yield and physiological responses of triticale and cowpea genotypes

Both triticale and cowpea genotypes did not significantly vary in transpiration rate (*E)*, stomatal conductance (*gs*), photosynthetic rate (*A*), the ratio of intercellular CO_2_ to atmospheric CO_2_ (*ci/ca*), instantaneous WUE (InstWUE), intrinsic WUE and carbon isotope composition (δ^13^C) (Table 2). Significant differences in genotypic performances were observed in biomass of cowpea and the grain yield of triticale. These findings are discussed in more detail in [15] for triticale and [16] for cowpea.

**Table 2:** Yield and physiological responses of triticale and cowpea genotypes.

Crop	Genotype	*E*	*gs*	*A*	*ci/ca*	*InsWUE*	*IntrWUE*	δ^13^C	Biomass	Grain yield
Triticale	Agbeacon	2.98	0.13	11.08	0.52	3.56	84.89	-26.84	8.60	2.55a
	Bacchus	2.96	0.12	11.79	0.40	4.08	118.91	-26.93	10.41	2.40a
	Rex	3.39	0.17	13.65	0.45	4.18	101.16	-26.94	9.48	2.33ab
	US2007	3.03	0.15	10.52	0.53	3.6	89.24	-27.23	9.08	1.25b
Cowpea	IT00K-529-1	1.88	0.068	9.41	0.32	4.86	166.18	-24.85	2.33b	0.87
	IT99K-1122	1.95	0.074	9.51	0.28	5.13	163.81	-25.13	2.65a	0.84
	TVu14632	1.93	0.076	10.32	0.28	5.13	168.46	-25.10	2.09b	0.83
	TVu4607	2.33	0.091	10.82	0.35	4.75	142.63	-24.82	3.38a	0.70

E: transpiration rate (mmol m^−2^ s^−1^); gs: stomatal conductance (mol m^−2^ s^−1^); A: photosynthetic rate (μmol m^−2^ s^−1^); ci/ca: ratio of intercellular CO_2_ and ambient CO_2_; WUEinst: instantaneous water use efficiency (μmol mmol^−1^); WUEintr: intrinsic water use efficiency (μmol mol^−1^); δ^13^C: carbon isotope composition. Different letters denote significant differences.

### 3.3 Yield of cowpea and triticale as influenced by moisture level

**Figure 1:**
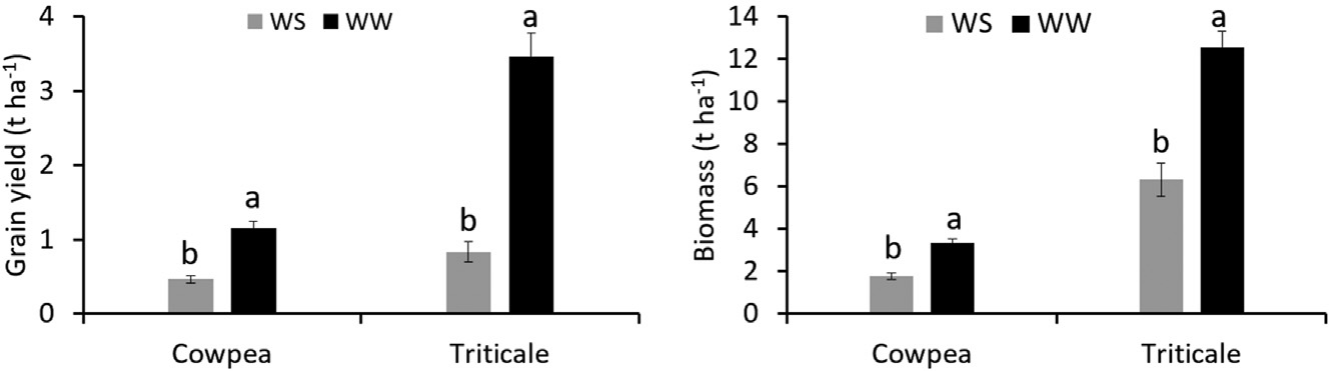
Grain yield and Biomass of cowpea and triticale as influenced by moisture level. ws: Water stressed ww: Well-watered.

Grain yield and biomass of both cowpea and triticale were significantly reduced by water stress (Figure 1). Grain yield and biomass were higher under WW compared to WS for both crops. A comparison of the significant losses observed in both cowpea and triticale showed that grain yield was more susceptible to drought compared to biomass. Water stress induced losses in grain yield of up to 75% in triticale and 60% in cowpea. These yield losses are congruent with the reported results of related studies [17, 18]. Bastos, Nascimento [17] observed similar cowpea grain yield losses of 60% due to water stress while Schittenhelm, Kraft [18] observed triticale grain yield losses of 65%. The reduction in biomass yield resulting from water stress was however lower than that observed with grain yield. The decrease in biomass yield between WW and SS was 47% and 48% for cowpea and triticale, respectively. It is however well known that biomass produced depends on the amount of water used. Biomass accumulation also depends on the amount of CO_2_ assimilated [19]. The results revealed that where the response to moisture level was significant, water-stressed conditions resulted in decreased photosynthetic rates and hence lower biomass (Table 3). Consequently, due to the low biomass produced, little assimilates may have been translocated for grain filling resulting in even lower grain yield. It is well established that under water stressed conditions, grain yield is not only dependent on biomass production but also on the proportion of biomass partitioning to grain [20].

**Table 3:** Effect of moisture level on leaf gas exchanges and carbon isotope composition.

Crop	Moisture Level	*Ci ppm*	*E mmol m*^*−2*^*s*^*−1*^	*gs* *mol m*^*−2*^*s*^*−1*^	*A* *μmol m*^*−2*^*s*^*−1*^	*ci/ca*	WUEintr μmol mol^−1^	WUEinst μmol mmol^−1^	δ^13^C (‰)
Cowpea	WS	88	1.45	0.04	7.34	0.22	193	5.29	-24.33
	WW	150	2.60	0.11	12.69	0.39	124	4.65	-25.61
		∗∗	∗∗	∗∗	∗	∗∗	∗∗∗	*ns*	∗∗∗
Triticale	WS	165	2.46	0.10	9.42	0.43	115	3.88	-25.83
	WW	201	3.82	0.19	14.44	0.53	77	3.79	-28.17
		∗	∗∗∗	∗∗∗	∗∗	∗	∗∗	ns	∗∗∗

Ci: intercellular CO_2_ concentration; E: transpiration rate (mmol m^−2^ s^−1^); gs: stomatal conductance (mol m^−2^ s^−1^); A: photosynthetic rate (μmol m^−2^ s^−1^); ci/ca: ratio of intercellular CO_2_ and ambient CO_2_; WUEinst: instantaneous water use efficiency (μmol mmol^−1^); WUEintr: intrinsic water use efficiency (μmol mol^−1^); δ^13^C: carbon isotope composition. Significance levels: ∗P < 0.05, ∗∗P < 0.01, ∗∗∗P < 0.001, ns means not significant.

### 3.4 Effect of moisture level on gas exchanges of cowpea and triticale

**Figure 2:**
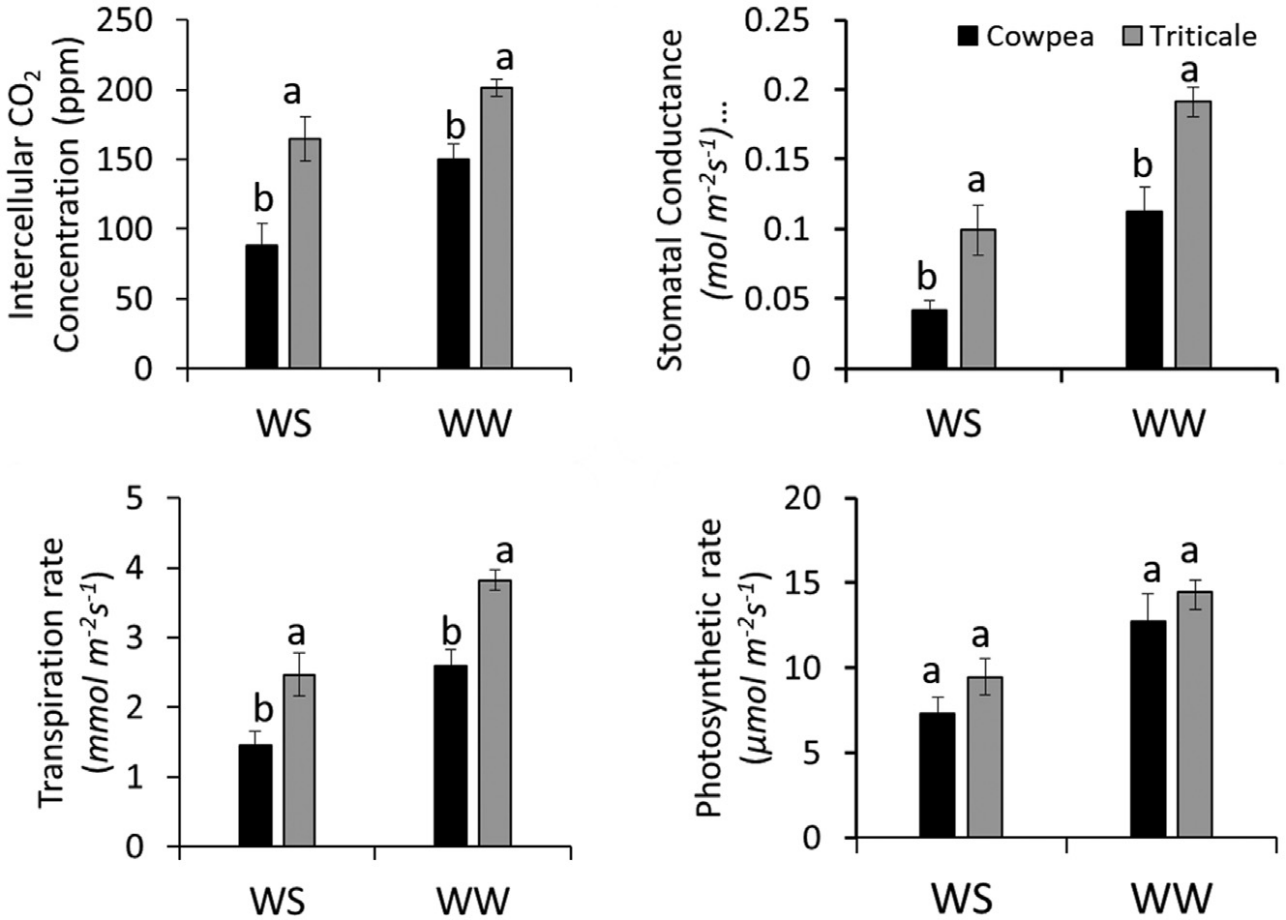
Transpiration rate, stomatal conductance, intercellular CO_2_, and photosynthetic rate of cowpea and triticale measured at flowering stage of each crop. ws: Water-stressed ww: Well-watered.

Here comparisons are made first on how moisture levels affected leaf gas exchanges of each crop (Table 3) followed comparison between the two C3 crops. The leaf gas exchanges of cowpea and triticale responded to moisture level (Table 3). Transpiration rate, *gs* and *A* were all low under WS conditions compared to under WW conditions. This effect of moisture level on gas exchanges was significant in both cowpea and triticale. The influence of moisture level on leaf gas exchanges has been reported in several different studies involving cowpea [2, 8, 21] and triticale [22, 23, 24]. A comparison of the two crops on how they performed in terms of the leaf gas exchanges, showed that triticale maintained higher *Ci*, *gs* and *E* compared to cowpea under both WS and WW (Figure 2). Triticale and cowpea did not differ in terms of photosynthesis rate even though triticale had relatively higher values compare to cowpea. Plausible reasons for such variations in leaf gas exchanges could be attributed to the variances in stomatal size and density or to differences in water extraction by the roots of the two crops [25]. Zhao, Sun [26], reported that drought increases stomatal density but decreases stomatal size and aperture which subsequently results in decreases in *A* and *E*. The associations in Tables 4 and 5 show highly significant positive relations between *gs* and *A;* and *gs* and *E* for all the two crops under both WW and WS confirming the strong influence of *gs* on *A* and *E* as reported in other studies [27].

**Table 4:** Association of variables in cowpea and triticale under WW.

Parameters	*ci*	*E*	*gs*	*A*	WUEintr	WUEinst	δ^13^C	Grain yield	Biomass
Cowpea									
*E*	-0.02								
*gs*	0.09	**0.96∗∗**							
*A*	-0.30	**0.94∗∗**	**0.92∗∗**						
*ci/ca*	**0.99∗∗**	-0.03	0.08	-0.30					
WUEintr	**-0.85∗∗**	-0.46	**-0.56∗**	-0.22					
WUEinst	**-0.70∗∗**	**0.65∗∗**	**0.61∗**	**0.84∗∗**	0.27				
δ^13^C	-0.44	**-0.51∗**	**-0.54∗**	-0.34	**0.69∗∗**	-0.06			
Grain yield	0.33	0.06	0.23	0.06	-0.36	0.02	**-0.60∗**		
Biomass	0.21	0.09	0.09	0.07	-0.22	-0.19	0.08	-0.08	
Triticale									
*E*	-0.13								
*gs*	0.17	**0.83∗∗**							
*A*	**-0.61∗**	**0.77∗∗**	**0.67∗∗**						
*ci/ca*	**0.99∗∗**	-0.12	0.19	**-0.60∗**					
WUEintr	**-0.91∗∗**	-0.23	**-0.54∗**	0.26					
WUEinst	**-0.78∗∗**	-0.08	-0.02	**0.57∗**	**0.69∗∗**				
δ^13^C	-0.12	-0.16	-0.20	-0.14	0.10	-0.02			
Grain yield	**-0.57∗**	0.18	0.00	0.48	**0.51∗∗**	**0.49∗**	0.05		
Biomass	0.08	-0.28	-0.29	-0.28	0.13	-0.04	0.21	0.23	

Bolded values are significant at P ≤ 0.05. Ci: intercellular CO_2_ concentration; E: transpiration rate (mmol m^−2^ s^−1^); gs: stomatal conductance (mol m^−2^ s^−1^); A: photosynthetic rate (μmol m^−2^ s^−1^); ci/ca: ratio of intercellular CO_2_ and ambient CO_2_; WUEinst: instantaneous water use efficiency (μmol mmol^−1^); WUEintr: intrinsic water use efficiency (μmol mol^−1^); δ^13^C: carbon isotope composition. Significance levels: ∗P < 0.05, ∗∗P < 0.01, ∗∗∗P < 0.001, ns means not significant.

**Table 5:** Association of variables in cowpea and triticale under WS.

Parameters	*ci*	*E*	*gs*	*A*	WUEintr	WUEinst	δ^13^C	Grain yield	Biomass
Cowpea									
*E*	0.42								
*gs*	0.44	**0.97∗∗**							
*A*	0.05	**0.89∗∗**	**0.88∗∗**						
*ci/ca*	**0.99∗∗**	0.39	0.41	0.02					
WUEintr	**-0.80∗∗**	**-0.65∗∗**	**0.69∗∗**	-0.32					
WUEinst	**-0.84∗∗**	-0.26	-0.26	-0.32	**0.73∗∗**				
δ^13^C	**0.64∗∗**	0.43	0.43	0.19	**-0.50∗**	**-0.68∗∗**			
Grain yield	0.38	0.25	0.25	0.22	-0.26	-0.27	0.27		
Biomass	0.18	-0.03	-0.03	-0.05	-0.26	-0.19	0.03	**0.57∗**	
Triticale									
*E*	0.35								
*gs*	0.49	**0.97∗∗**							
*A*	0.03	**0.89∗∗**	**0.83∗∗**						
*ci/ca*	**1∗∗**	0.36	0.49	0.03					
WUEintr	**-0.95∗∗**	**-0.57∗**	**-0.68∗∗**	-0.29					
WUEinst	**-0.89∗∗**	-0.14	-0.25	0.27	**0.78∗∗**				
δ^13^C	**-0.55∗**	**-0.81∗∗**	**-0.82∗∗**	**-0.62∗**	**0.68∗∗**	0.34			
Grain yield	0.16	0.26	0.21	0.26	-0.28	-0.06	-0.33		
Biomass	0.44	**0.67∗∗**	**0.64∗∗**	0.43	**-0.54∗**	-0.42	**-0.78∗**	0.25	

Note: Bolded values are significant at P ≤ 0.05. Ci: intercellular CO_2_ concentration; E: transpiration rate (mmol m^−2^ s^−1^); gs: stomatal conductance (mol m^−2^ s^−1^); A: photosynthetic rate (μmol m^−2^ s^−1^); ci/ca: ratio of intercellular CO_2_ and ambient CO_2_; WUEinst: instantaneous water use efficiency (μmol mmol^−1^); WUEintr: intrinsic water use efficiency (μmol mol^−1^); δ^13^C: carbon isotope. Significance levels: ∗P < 0.05, ∗∗P < 0.01, ∗∗∗P < 0.001, ns means not significant.

### 3.5 Effect of moisture level on ci/ca, δ^13^C, WUEintr and WUEinst of cowpea and triticale

**Figure 3:**
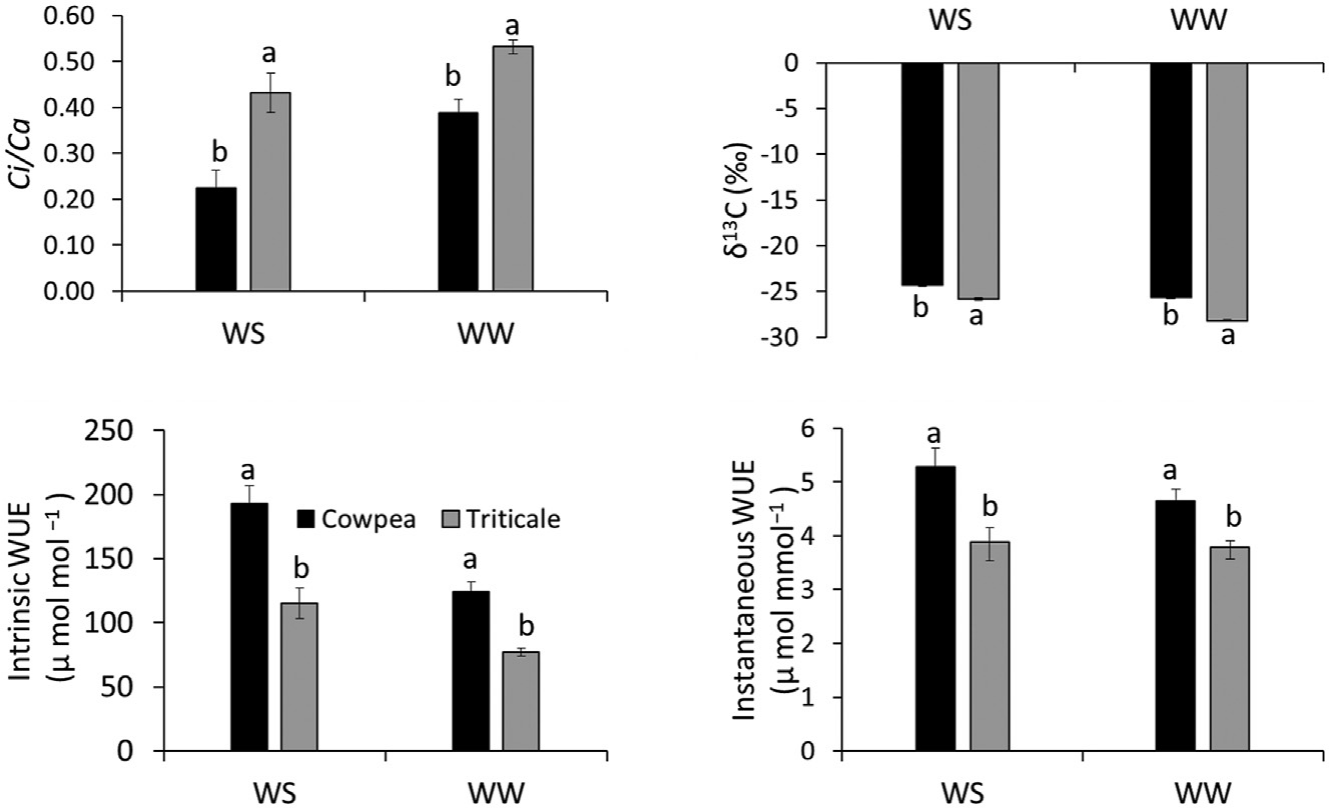
Ratio of intercellular CO_2_ to ambient CO_2_ (*ci/ca*) carbon isotope composition (δ^13^C), WUEintr and WUEinst of cowpea and triticale measured at flowering stage of each crop. ws: Water-stressed ww: Well-watered.

The *ci/ca*, δ^13^C, and WUEintr, all responded to differences in moisture level in both cowpea and triticale (Table 3). However, WUEinst was not influenced by water stress in both crops while WUEintr was greater under WS compared to WW conditions. In both crops, WUEintr was approximately 50% higher under WS compared to WW. Water stress (WS) reduced *ci/ca* compared to WW in both cowpea and triticale. Also, there was higher ^13^C discrimination under WW leading to more negative δ^13^C values under WW in both crops. The *ci/ca* has been described by some authors as a major factor determining ^13^C discrimination in plants [28]. Intercellular CO_2_ concentration, as well as *ci/ca,* were all significantly lower in cowpea compared to triticale under both WW and WS conditions. These differences were also reflected in the δ^13^C values where cowpea discriminated ^13^C less resulting in less negative δ^13^C values compared to triticale. As alluded to earlier, Figure 2 shows significantly higher *gs* in triticale than cowpea but did not show any difference in *A*. A lower *gs* in cowpea accompanied by a relatively higher *A* resulted in less ^13^C discrimination by Ribulose bisphosphate carboxylase/oxygenase in cowpea and hence less negative δ^13^C values [29]. When comparing the performance of the two crops, it was observed that both crops differed in all the four parameters shown in Figure 3. The *ci/ca* was higher in triticale compared to cowpea thus resulting in more negative δ^13^C values in triticale compared to cowpea. On the other hand, cowpea performed better than triticale in both WUEintr and WUEinst and this was observed for both WW and WS conditions.

Responses of leaf gas exchanges to water stress are known to be species and genotype specific. However, rates of decrease in *gs* due to water stress as well as the time to recover are different even among and C3 crops [12, 13]. In this study, a comparison was made between two C3 crops, both of which are regarded as drought tolerant. Despite their drought tolerance, they behaved differently different. Triticale maintained relatively higher leaf gas exchanges than cowpea which also influenced δ^13^C. However, cowpea had better leaf level WUE (WUEintr and WUEinst). In addition, the relationship between *ci/ca* and δ^13^C was negative under WW conditions for all the crops even though not significant. This shows that there is more ^13^C discrimination under high *ci* values. However, under WS conditions, contrasting results were observed. Cowpea showed a significant positive relationship between *ci/ca* and δ^13^C while triticale showed negative relationships.

In conclusion, the findings of this study reveal a significant influence of water availability on both biomass and grain yield. It was hypothesized that the two C3 crops, cowpea and triticale would not be expected to vary in leaf gas exchange and carbon isotope composition. However, the study highlighted differences in C3 crops’ leaf gas exchange response to water availability.

It also revealed the differences in grain yield sensitivity to water stress between triticale and cowpea.

## Declarations

### Author contribution statement

Lawrence Munjonji: Conceived and designed the experiments; Performed the experiments; Analyzed and interpreted the data; Wrote the paper.

Kingsley Kwabena Ayisi: Conceived and designed the experiments; Contributed reagents, materials, analysis tools or data.

### Funding statement

This study was funded by VLIR IUC Project and the Risk and Vulnerability Science Center at the University of Limpopo..

### Data availability statement

Data included in article/supplementary material/referenced in article.

### Declaration of interests statement

The authors declare no conflict of interest.

### Additional information

No additional information is available for this paper.
